# Maturation, inactivation, and recovery mechanisms of soluble guanylyl cyclase

**DOI:** 10.1016/j.jbc.2021.100336

**Published:** 2021-01-26

**Authors:** Dennis J. Stuehr, Saurav Misra, Yue Dai, Arnab Ghosh

**Affiliations:** 1 Department of Inflammation and Immunity, Lerner Research Institute, Cleveland Clinic, Cleveland, Ohio, USA; 2 Department of Biochemistry and Molecular Biophysics, Kansas State University, Manhattan Kansas, USA

**Keywords:** Hsp90, cytochrome b5 reductase, nitric oxide, protein oxidation, protein nitrosation, cell signaling, hypertension, CC, coiled-coil, HDX-MS, hydrogen-deuterium exchange mass spectrometry, NO, nitric oxide, PAS, Per-Arnt-Sim, sGC, soluble guanylate cyclase

## Abstract

Soluble guanylate cyclase (sGC) is a heme-containing heterodimeric enzyme that generates many molecules of cGMP in response to its ligand nitric oxide (NO); sGC thereby acts as an amplifier in NO-driven biological signaling cascades. Because sGC helps regulate the cardiovascular, neuronal, and gastrointestinal systems through its cGMP production, boosting sGC activity and preventing or reversing sGC inactivation are important therapeutic and pharmacologic goals. Work over the last two decades is uncovering the processes by which sGC matures to become functional, how sGC is inactivated, and how sGC is rescued from damage. A diverse group of small molecules and proteins have been implicated in these processes, including NO itself, reactive oxygen species, cellular heme, cell chaperone Hsp90, and various redox enzymes as well as pharmacologic sGC agonists. This review highlights their participation and provides an update on the processes that enable sGC maturation, drive its inactivation, or assist in its recovery in various settings within the cell, in hopes of reaching a better understanding of how sGC function is regulated in health and disease.

Soluble guanylyl cyclase (sGC, EC 4.6.1.2) generates cGMP in response to nitric oxide (NO) (1, 2, 3, 4, 5). Interest in sGC centers on at least four aspects: NO-driven generation of the important second messenger molecule, cGMP, makes sGC the essential middle component of intracellular NO- and cGMP-driven signaling cascades; the molecular mechanism by which NO activates sGC is still not completely understood and involves intriguing relationships between hemeprotein structure and function; sGC is also representative of a broad family of heme proteins that are expressed throughout biology to sense and respond to environmental O_2_ or NO; and finally, in mammals, sGC dysfunction contributes to the pathogenesis of several cardiovascular, pulmonary, and autoimmune diseases (6, 7, 8, 9, 10). Thus, boosting sGC activity or preventing or reversing its inactivation are important therapeutic and pharmacologic goals (11, 12, 13).

This review provides an update on molecular events and mechanisms that enable sGC maturation, drive sGC inactivation, or potentially assist in sGC recovery in various settings within the cell. We highlight the involvement of molecules and proteins implicated in these processes, including reactive oxygen species, NO, cellular heme, cell chaperones and redox proteins, as well as pharmacologic agonists. We encourage readers interested in biomedical aspects of sGC, the molecular mechanisms of sGC catalysis and activation, or sGC-like proteins found in lower organisms to consult recent reviews and reports that focus specifically on these topics (13, 14, 15, 16, 17, 18, 19, 20, 21).

## General properties of sGC

**Figure 1:**
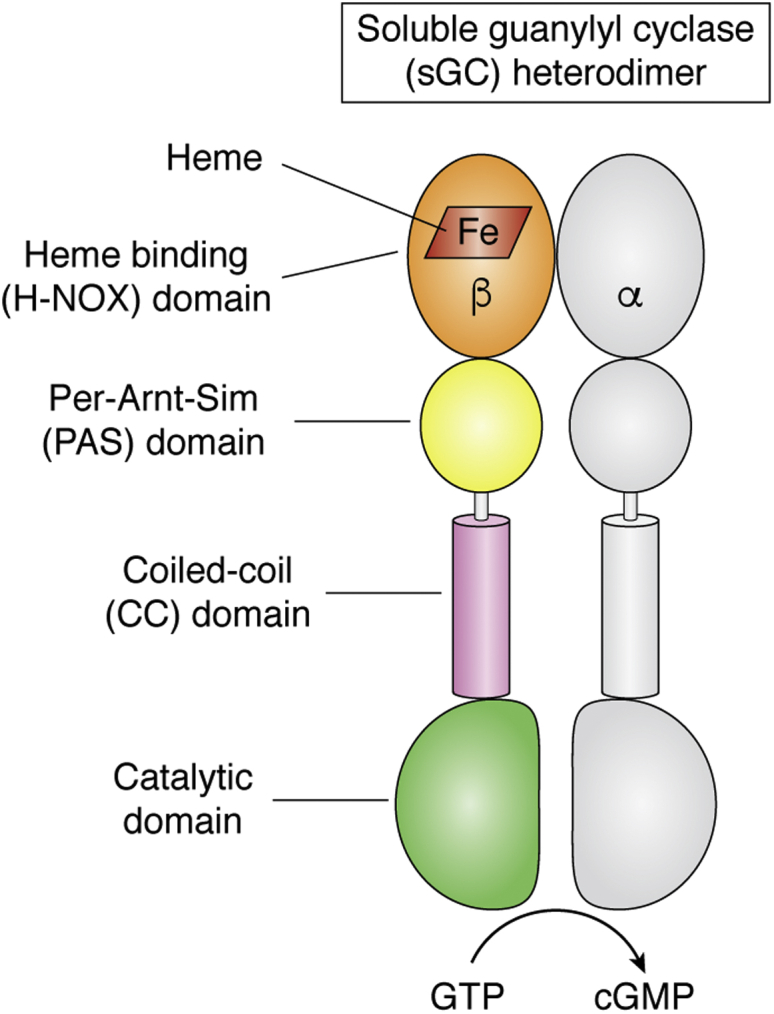
**The sGC heterodimer.** The protein domains are noted, and heme is shown as a *red parallelogram*.

sGC generates cGMP in its basal state but gains far higher activity when its heme binds NO (14). Figure 1 illustrates the quaternary structure, domain organization, and heme binding site of sGC. The mature and functional sGC is a heterodimer of alpha and beta subunits. Although gene and splice variants of sGC subunits are expressed in vertebrates (22, 23), the predominant sGC in most circumstances consists of an α1 and β1 heterodimer (14, 22, 23, 24, 25). sGC maturation involves posttranslational heme insertion into the apo-sGCβ subunit, followed by binding of sGCβ to sGCα to form the sGC heterodimer (26). The heterodimer interface primarily consists of surface contacts formed between the respective Per-Arnt-Sim (PAS) domains, the coiled-coil (CC) domains, and catalytic domains (18, 27) of the respective subunits. In addition, cross-subunit interactions between different domains can form that likely further stabilize and modify the heterodimer structure (18, 20, 28). Only the ferrous heme-containing sGCαβ heterodimer can respond to NO to catalyze cGMP formation (14, 18, 29). NO binding to the ferrous heme of sGCβ breaks the proximal His-heme iron bond (14, 18), initiating a series of structural changes that project through the heterodimer CC domains to the catalytic domains, resulting in an increased cGMP synthesis activity (17, 20, 30). If the sGC heme becomes oxidized to the ferric state, it is no longer activated by NO and is also more prone to dissociate from the sGC (18, 31, 32, 33, 34, 35, 36). Thus, to function in NO-cGMP signaling cascades, sGCβ must acquire heme, form a heterodimer with sGCα, and maintain its heme in the ferrous state.

## sGC maturation to a heterodimer

The requirement to form a heterodimer places an added challenge on sGC maturation. A number of factors may impact the process. The sGC α and β subunits are encoded by separate genes, and the extent to which their transcription is coordinated is unclear (37). When sGC α and β subunits were expressed separately in eukaryotic cells, they formed homodimers, although they did so to a much lesser extent when the two different subunits were co-expressed (38). Predominant sGCβ or sGCα expression appears to take place in certain cells or tissues (39, 40, 41, 42, 43), and both sGC subunits can display reduced buildup under stress conditions (44, 45, 46, 47, 48, 49). Finally, although many H-NOX protein family members have attached catalytic domains (50, 51) they typically function as monomers or as homodimers rather than heterodimers (15, 21, 51), distinguishing sGC in this respect.

### Immature sGCβ associates with chaperone Hsp90

**Figure 2:**
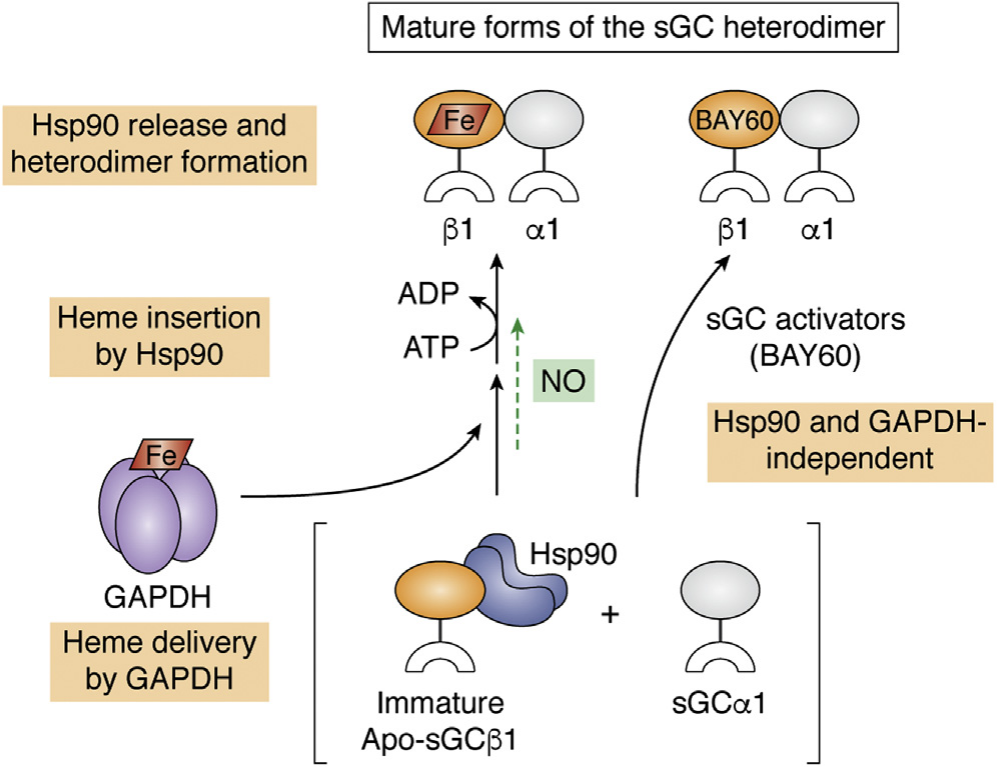
**Maturation of the sGC heterodimer.** Steps in maturation start from the bottom of the Figure and continue upward. For simplicity, the sGC subunits are depicted as *orange* or *gray ovals* representing the H-NOX plus PAS domains attached to a *white crescent* that represents their CC and catalytic domains. Following translation, the immature apo-sGCβ1 subunit forms a complex with Hsp90 and does not associate with an sGCα1 subunit. GAPDH delivers heme (*red parallelogram*), which is inserted into the apo-sGCβ1 in an ATP-driven, Hsp90-dependent process. Heme insertion triggers Hsp90 dissociation from sGCβ1, and this allows it to bind an sGCα1 subunit to form the mature sGC heterodimer. Low levels of NO (*green*) can stimulate the maturation process in an as yet undefined way (*green arrow*). sGC activator compounds like BAY 60 can bind within immature apo-sGCβ1 and drive maturation to the sGC heterodimer independent of Hsp90, GAPDH, or heme. CC, coiled-coil; PAS, Per-Arnt-Sim; sGC, soluble guanylate cyclase.

After translation the sGCβ subunit is in a heme-free state (apo-sGCβ). Apo-sGCβ is stable and can accumulate in mammalian cells; cells that are made heme-deficient exclusively accumulate apo-sGCβ (52). Importantly, apo-sGCβ associates with the major cytosolic chaperone Hsp90 instead of with an sGCα subunit partner (52, 53) (Fig. 2).

Hsp90 is a cytosolic chaperone that controls the maturation events and lifetimes of numerous protein clients (54, 55, 56, 57). Hsp90 interacts directly with substrate proteins and utilizes both its inherent ATPase activity and recruitment of partner co-chaperone proteins to stabilize a client protein and direct its final folding events. Hsp90 can also govern the chaperoned client's interactions with protein partners. Apo-sGCβ is one of several heme proteins that associate with Hsp90 in their heme-free forms in mammalian cells and that require Hsp90 for their maturation, the others being NO synthases (58), hemoglobins β and γ (59), and myoglobin (60). Interestingly, the Hsp90-sGC association was first discovered in the context of its stabilizing sGC and increasing cGMP synthesis (61, 62, 63). It was thus assumed that Hsp90 chaperones the sGC heterodimer. However, recent evidence (discussed below) suggests that these changes instead probably reflect the ability of Hsp90 to drive maturation of the immature apo-sGCβ subunit. The molecular features and implications of Hsp90 interactions with apo-sGCβ are fundamentally important and are discussed throughout this review.

### Cells and tissues contain significant levels of immature sGC

It is widely assumed that healthy cells and tissues express sGC predominantly or even exclusively in its mature, ferrous heme-containing heterodimeric form. This assumption appears to be incorrect, as judged from experiments with small-molecule sGC agonists that either activate only the mature ferrous sGC heterodimer (*i.e.*, BAY 41-2272, hereafter called BAY 41; Riociguat; IWP-051) or that activate only the heme-free or heme-oxidized forms of sGC (*i.e.*, BAY 60–2270, hereafter called BAY 60; Cinaciguat; BAY 58-2667, hereafter called BAY 58) (64). These agonists provided a means to assess the relative levels of the different sGC forms present in cells or tissues. They have been utilized in studies with human umbilical vein endothelial cells, primary human airway smooth muscle cells, a cell line that naturally expresses sGC (RFL6), transfected COS-7 or HEK cells, and tissues from a number of animal disease models (53, 65, 66, 67, 68, 69). In all cases, results with the pharmacologic agonists indicated that heme-free or heme-oxidized forms of sGC were present.

Antibody pull-down and Western analyses have also independently assessed the relative levels of sGCβ that is present as part of an sGC heterodimer *versus* as a dissociated sGCβ subunit in complex with Hsp90 (53, 65, 70). Notably, the first tissue found to naturally display a low proportion of sGC heterodimer by these means was the cerebrum of adult rats (71) and most recently is the airway smooth muscle of severe asthmatics (72).Together, the pharmacologic and biochemical studies establish that cells and tissues invariably contain a mix of mature heterodimer and immature sGC forms, with the immature form comprising a significant fraction of the total sGC, approximately 20 to 60% of total (26).

In addition to challenging the historical view that sGC is primarily or exclusively present as a mature heterodimer, this concept has important physiological implications. Specifically, it suggests that stimulating the existing pool of immature apo-sGCβ toward heme insertion and heterodimer formation could provide an alternative way to significantly increase sGC activity in cells and tissues independent of any gene activation or new protein synthesis. How sGC heterodimer formation occurs and is stimulated by natural mechanisms or pharmacologic agonists is discussed in the following sections.

### sGC heme delivery and insertion involves GAPDH and Hsp90

In mammalian cells the heme destined for apo-sGCβ is provided by GAPDH (70) (Fig. 2). GAPDH binds mitochondrially derived heme (73, 74) and delivers it to apo-sGCβ and to other cellular hemeproteins during their maturation (73, 74, 75). Both free and heme-loaded GAPDH bind to apo-sGCβ but not to heme-replete sGCβ, and a GAPDH–heme complex transfers its heme to apo-sGCβ within 2 min (70). These observations suggest that heme delivery to apo-sGC may involve a direct heme transfer from GAPDH, but the details remain to be determined.

In mammalian cells heme insertion into apo-sGCβ also depends on Hsp90. Heme insertion requires that Hsp90 has intact ATP-ase activity (52) and relies on direct interaction between Hsp90 and apo-sGCβ (76) (Fig. 2). Studies indicate that specific regions in the PAS domain and adjacent CC domain linker of apo-sGCβ interact with the middle domain of Hsp90 (76, 77). These interactions are essential for heme insertion into apo-sGCβ in mammalian cells (76, 77). The Hsp90 interaction with apo-sGCβ appears to be selective because Hsp90 does not interact with the similar PAS and CC domains of sGCα (52, 77).

Notably, Hsp90 also interacts with certain other client proteins through their PAS domains (78, 79, 80, 81). The Hsp90–PAS interactions share common features; they all control small ligand binding to the client protein (in the case of sGC, heme insertion into the apo-sGCβ) and control the client's ability to interact with a partner subunit or partner protein to form a mature species (in the case of sGC, the interaction of the sGCβ subunit with the sGCα subunit to form a heterodimer). A molecular model for the Hsp90–apo-sGCβ complex has been proposed (77). How this complex governs the sGCβ–sGCα interaction and may facilitate heme insertion is discussed in Molecular insights into Hsp90-driven sGC maturation of this review.

### The Hsp90 interaction insures that functional sGC forms in cells

Hsp90 dissociates from apo-sGCβ only after the latter receives heme (53) (Fig. 2), likely in response to structural changes that take place in sGCβ upon its heme insertion (76). The Hsp90 association and dissociation events are coordinated with sGCβ heme insertion in such a way as to guide sGC maturation. First, Hsp90 association with apo-sGCβ prevents apo-sGCβ from binding to a sGCα subunit. Indeed, association between sGCα and apo-sGCβ subunits only takes place in cells under circumstances in which Hsp90 cannot bind to the apo-sGCβ, (53, 76). Second, restricting Hsp90 dissociation to occur only after heme insertion is finished ensures that only heme-replete sGCβ is available to partner with sGCα. Thus, consecutive Hsp90 association and dissociation events prevent the formation of heme-free nonfunctional sGC heterodimers, while simultaneously ensuring that heme-replete and functional sGC heterodimers form in cells (Fig. 2).

Several synthetic sGC agonists bind within the empty heme pocket of apo-sGCβ as part of their mechanism of activation (*e.g.*, BAY 58 and BAY 60). Interestingly, these agonists do so without requiring assistance from Hsp90. Moreover, once bound, these agonists can induce Hsp90 to dissociate from sGCβ in mammalian cells (Fig. 2) (53). Crystal structures of the *Manduca sexta* sGC–like protein containing BAY 58, BAY 60, or similar agonists highlight the structural changes that occur as a consequence of their binding (82, 83). How these structural changes overlap with those that induce Hsp90 release from sGCβ is an important question for further research.

### The curious case of NO

NO impacts sGC maturation and function in two ways that are distinct from its ability to activate the mature sGC heterodimer by binding to its ferrous heme (14). The two other impacts of NO are functionally opposed and depend on the flux and duration of NO production and the extent of its accumulation in the system. At lower levels of production and accumulation, NO exerts a positive effect by promoting sGC maturation (Fig. 2). At higher levels, NO exerts a negative effect and inactivates the sGC heterodimer. These two actions of NO are mechanistically distinct and are discussed in separate sections below.

### NO can drive sGC maturation

Relatively low levels of NO trigger maturation of the immature apo-sGCβ population within cells (Fig. 2). For example, when sGC-expressing cells are exposed to a slow-release NO donor, their apo-sGCβ population switched association from Hsp90 to sGCα, increasing the proportion of NO-and BAY 41-responsive (*i.e.*, ferrous heme-containing) sGC heterodimer (53). This NO-driven maturation depended on Hsp90 and also relied upon a sufficiently high cellular heme content. The same phenomenon was also observed in a co-culture system, in which NO synthase–expressing cells cultured in permeable inserts were placed over a layer of rat fibroblast cells (RFL-6) that natively express sGC. NO released by the insert cells increased sGCα–sGCβ association in the RFL-6 cells within the first 2 h (65). The increase in sGC heterodimer coincided with a shift in sGC pharmacologic response. In particular, cGMP production increased in response to BAY 41, which only activates the mature heterodimer and decreased in response to BAY 60, which only activates heme-free or heme-oxidized sGC. These and related findings (53) led to the proposal that NO drives sGC maturation by stimulating heme insertion into the cellular pool of apo-sGCβ. However, further research is needed to determine whether this is how NO actually drives sGC maturation and under what physiological conditions NO may do so. Regardless, the ability of low NO levels to promote maturation of immature and nonfunctional sGC protomers that are naturally present in cells and tissues (*i.e.*, apo-sGCβ) is of major importance. In particular, NO-driven sGC maturation presents a means by which cells and tissues can increase their level of functional sGC, independent of changes in sGC gene activation, sGC protein synthesis, or stability.

### Pharmacologic agonists can drive sGC maturation

sGC activators like BAY 60 can also drive maturation of the apo-sGCβ population in cells (52). However, unlike NO-driven maturation, BAY 60 binding causes Hsp90 to dissociate from apo-sGCβ, and its ability to drive maturation shows no dependence on Hsp90 or on cell heme content. This implies that agonists like BAY 60 bind within the heme pocket of the cellular apo-sGCβ pool to drive sGC maturation without any assistance from GAPDH or Hsp90, which normally participate in native maturation of sGC (Fig. 2). The remarkable ability of sGC activators to do this deserves better recognition and further study.

## Molecular insights into Hsp90-driven sGC maturation

**Figure 3:**
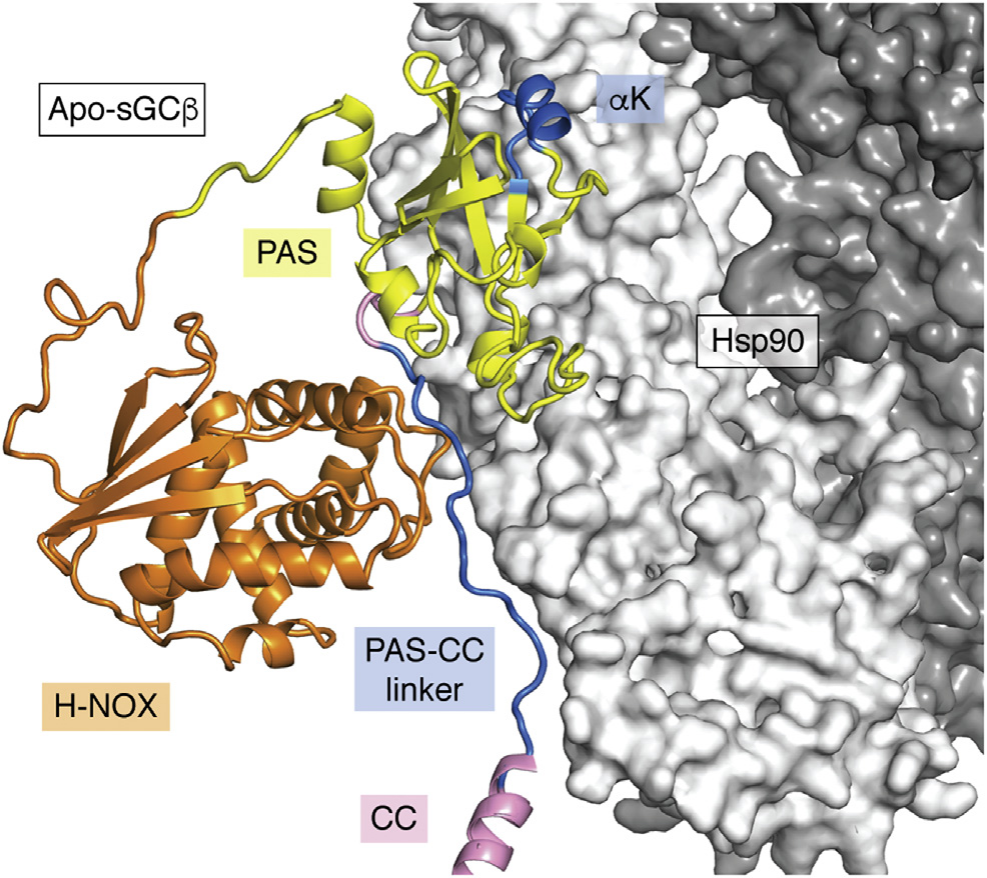
**Chaperoning of apo-sGCβ by Hsp90.** The model of the apo-sGCβ–Hsp90 complex was generated as described (76). The Hsp90 dimer is colored *light/dark gray*. The H-NOX, PAS, linker, and coiled-coil domains of apo-sGCβ are colored *orange*, *yellow*, *pink*, and *pink*, respectively, while segments that directly contact Hsp90 as judged by HDX-MS, and other experimental data are colored *blue* and separately labeled (helix αK and a portion of the PAS-CC linker). CC, coiled-coil; HDX-MS, hydrogen-deuterium exchange mass spectrometry; PAS, Per-Arnt-Sim; sGC, soluble guanylate cyclase.

Structural models of the sGCβ–Hsp90 complex have been built based on biophysical studies and application of Hydrogen-Deuterium exchange mass spectrometry (HDX) to the complex of truncated apo-sGCβ (residues 1–359) and the Hsp90-M domain, which were shown to mediate the interactions between the corresponding full-length proteins (77). Figure 3 depicts a representative molecular model that shows apo-sGCβ in complex with a closed (nucleotide-bound) form of the Hsp90 dimer (77). Two specific epitopes in apo-sGCβ shown to interact with Hsp90 are highlighted in blue. These consist of a surface-exposed alpha helix in the PAS domain (αK; secondary structure designations as presented in Kang *et al*. [17]) and a structurally dynamic segment of approximately 19 residues that links the PAS domain with the CC domain (αL-αM; PAS-CC linker). The complementary interacting patches in Hsp90 are not shown in Figure 3 but are located on its M domain and at the juncture of its M and C domains (76, 77).

Recently, the accuracy and biological relevance of the model complex was tested by incorporating mutations and deletions into apo-sGCβ that were predicted by the model to disrupt sGCβ–Hsp90 interaction (76). Most of these structure-based modifications either weakened or prevented complex formation between purified apo-sGCβ and Hsp90 *in vitro*, as well as when the proteins were expressed in mammalian cells (76). The study further showed that heme insertion requires Hsp90 to bind directly to apo-sGCβ in cells. In sum, the study supported the biological relevance of the Hsp90–apo-sGCβ model complex and confirmed that the principal mode of association involves a bipartite distributed interaction between the M domain of Hsp90 with the PAS domain and PAS-CC linker elements of apo-sGCβ.

Cryo-EM structures of the full-length sGC heterodimer in both its NO-free and NO-bound activated states have also become available (17, 20). These structures allow us to discern how the conformation of sGCβ may differ when it is in the sGC heterodimer *versus* in the Hsp90–apo-sGCβ complex and thus gain insight into how Hsp90 binding may enable heme insertion during sGC maturation.

### What enables sGCβ to interact with Hsp90 and sGCα on a mutually exclusive basis?

**Figure 4:**
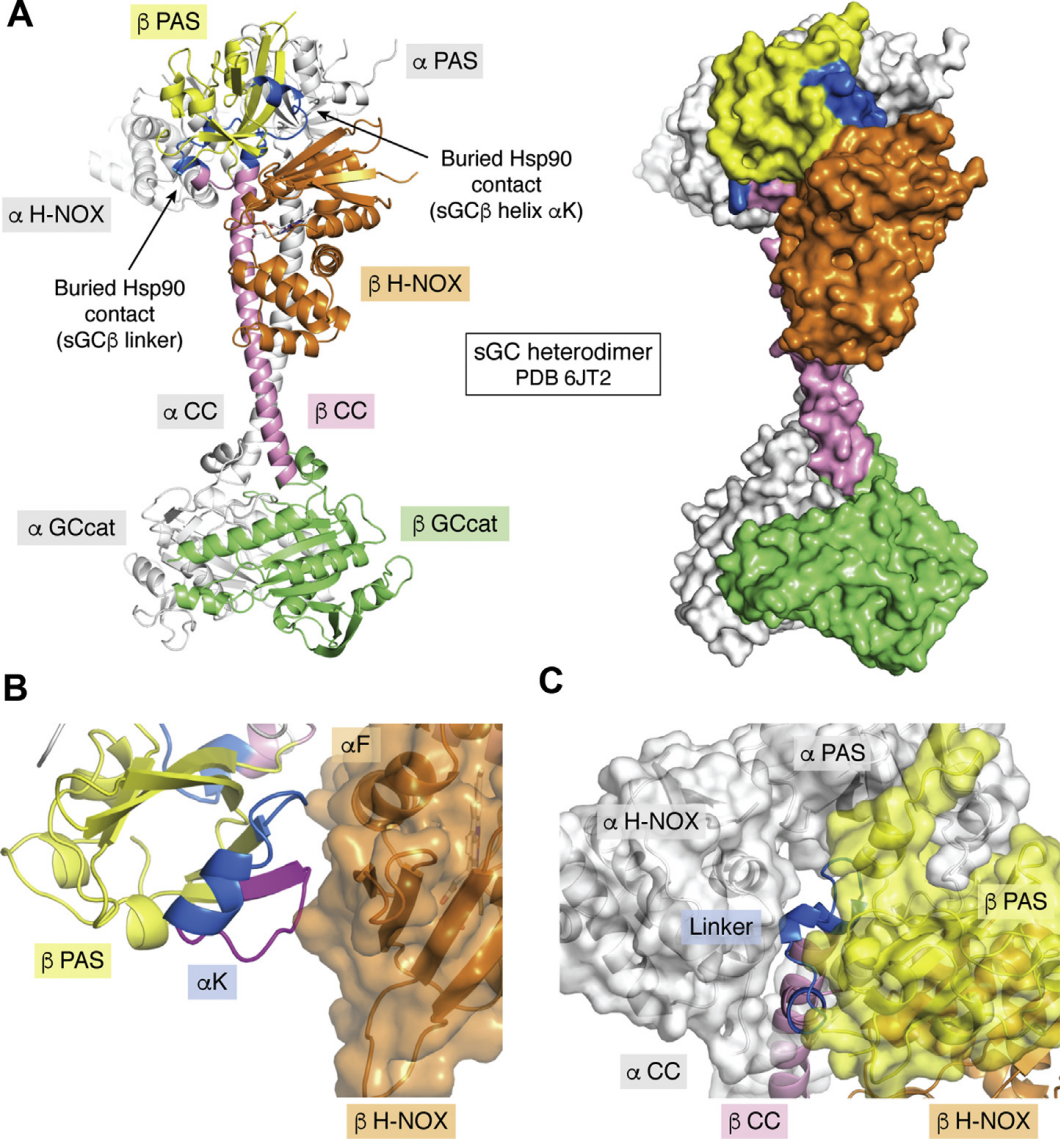
**Structure of the sGC heterodimer shows occlusion of the sGCβ Hsp90-interacting sites.***A*, the sGC heterodimer in the NO-bound state (PDB ID 6jt2; Kang *et al*. [17]) is shown in cartoon and surface presentations. The H-NOX, PAS, linker, coiled-coil, and catalytic domains of sGCβ are colored *orange*, *yellow*, *pink*, *pink*, and *green*, respectively. The entirety of sGCα is colored *white*. Segments of the sGCβ PAS and linker domains that interact directly with Hsp90 are colored *blue* and labeled. *B*, close-up showing the intradomain packing between the H-NOX domain and the Hsp90 binding segment around helix αK in the heterodimer. A segment near helix αK that exhibits increased solvent exposure when Hsp90 binds to apo-sGCβ is colored *magenta*. *C*, close-up showing how the Hsp90 binding segment within the sGCβ PAS-CC linker is buried at the interfaces of the sGCα H-NOX and the PAS and CC domains of sGCβ in the sGC heterodimer. CC, coiled-coil; NO, nitric oxide; PAS, Per-Arnt-Sim; sGC, soluble guanylate cyclase.

Apo-sGCβ interacts with Hsp90 and sGCα on a mutually exclusive basis (26, 76). This allows cells to maximize formation of heme-containing functional sGC heterodimers while minimizing formation of heme-free nonfunctional sGC heterodimers. But what factors enforce this mutual exclusion of sGCβ binding partners? A comparison of the sGC heterodimer structures with modeled Hsp90–apo-sGCβ complexes reveals that key structural elements in sGCβ play dual and mutually exclusive roles in the two different contexts. Helix αK and nearby residues in the sGCβ PAS domain mediate the direct interaction of apo-sGCβ with Hsp90 (Fig. 3). However, these binding determinants are largely buried in the sGC heterodimer structures. They participate instead in intramolecular packing interactions with the H-NOX domain of sGCβ, near its functionally critical αF helix (Fig. 4, *A* and *B*). Similarly, the PAS-CC linker (αL-αM) interacts with Hsp90 in apo-sGCβ (Fig. 3) but is mostly buried in the sGC heterodimer structures. In the latter, the linker sequence bridges an intermolecular interaction between the sGCα–HNOX and the sGCβ–PAS domains (Fig. 4, *A* and *C*). Additionally, the PAS-CC linker mediates interactions between the CC domain of sGCβ and its sGCα CC domain counterpart (Fig. 4, *A* and *C*). Thus, structural elements that are utilized by apo-sGCβ to interact with Hsp90 instead mediate important intramolecular and intermolecular interactions within the sGC heterodimer. Interactions of helix αK and the PAS-CC linker elements with Hsp90 sterically preclude the interactions they make within the sGC heterodimer and vice versa. These structural constraints explain how sGCβ interactions with Hsp90 and sGCα remain mutually exclusive.

### How might Hsp90 promote heme insertion into apo-sGCβ?

**Figure 5:**
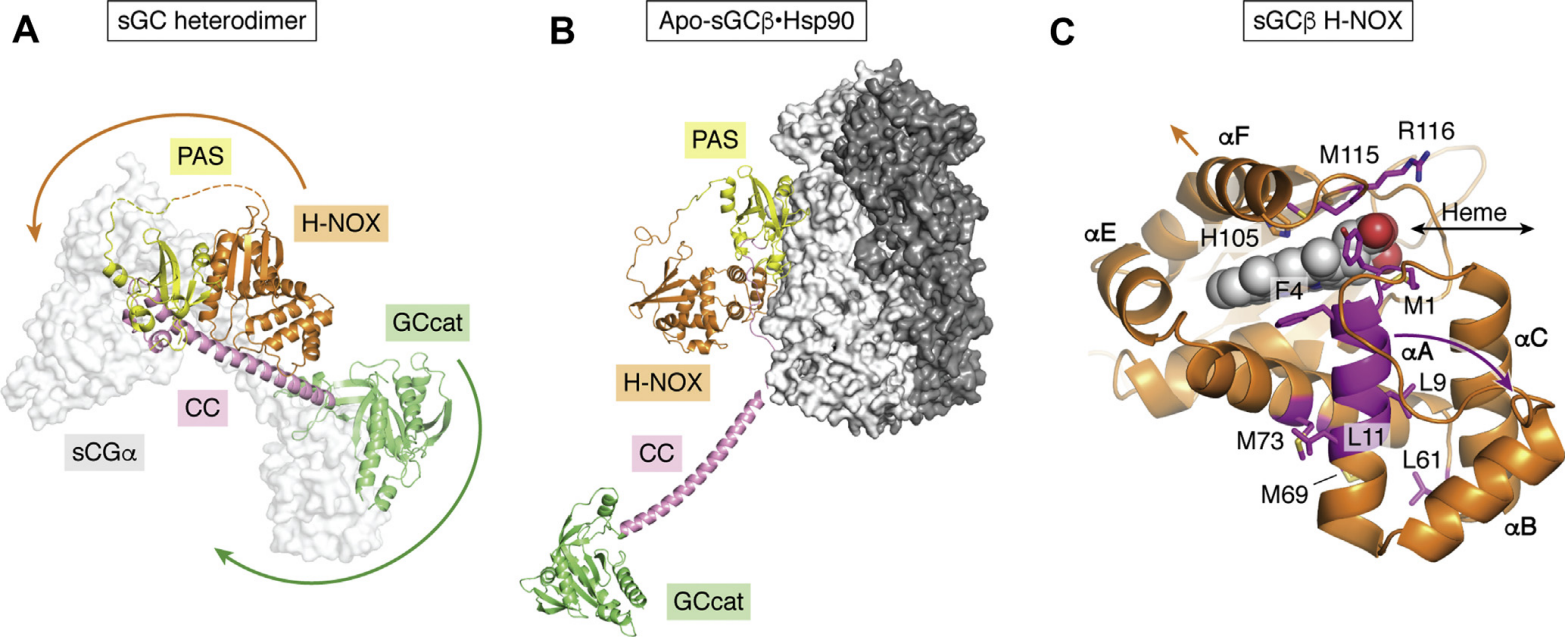
**Hsp90 stabilizes a structurally open conformation of apo-sGCβ to enable heme insertion.***A* and *B*, conformations of the sGCβ subunit when it is in the sGC heterodimer (PDB ID 6jt2; Kang *et al*. [17]) *versus* in complex with Hsp90 (docking model, Dai *et al.* [76]). The *yellow* PAS domain is kept in an identical orientation in both panels. The *arrows* indicate movements of sGCβ domains that must occur relative to the *yellow* PAS domain in order to allow Hsp90 binding, thus creating a more open structure with a free H-NOX domain. *C*, closeup of the sGCβ H-NOX domain when present in the sGC heterodimer (PDB 6jt0; Kang *et al*. [17]) with the bound heme shown in *sphere* representation. The N-terminal αA helix and selected residues colored in *magenta* undergo deprotection when Hsp90 binds to apo-sGCβ. The *magenta* and *orange arrows* indicate potential Hsp90-driven movements of helices αA, αB, αC, and αF away from the rest of the H-NOX domain that increase exposure of the heme pocket to enable heme delivery. The *dark gray arrow* indicates a potential heme entry pathway. NO, nitric oxide; PAS, Per-Arnt-Sim; sGC, soluble guanylate cyclase.

Hsp90 binding typically influences the conformation of its client proteins (57). Accordingly, the HDX data, modeling, and EM structures together suggest that Hsp90 binding forces apo-sGCβ to adopt a more extended conformation compared with when it is on its own in solution or when it is in the heterodimer (Fig. 5, *A* and *B*). Hsp90 binding would sterically prevent intramolecular contacts forming between the sGCβ H-NOX, PAS, and CC domains that are otherwise present in the heterodimer structures. The evidence also suggests that similar intramolecular contacts form in apo-sGCβ when it is left to itself. For example, an sGCβ PAS domain loop that is located near helix αK and makes an intramolecular interaction with the sGCβ H-NOX domain in the heterodimer becomes more solvent-exposed when Hsp90 binds to apo-sGCβ (Fig. 4*B*) (77). This implies that the intramolecular interaction is present in (unpartnered) apo-sGCβ and that Hsp90 binding disrupts it. Together, these considerations and the modeling indicate that the H-NOX domain cannot maintain its intermolecular interactions within apo-sGCβ when it is bound to Hsp90 and becomes free to swing away from the PAS domain (Fig. 5, *A* and *B*).

The structural opening that is driven by Hsp90 is likely to facilitate heme insertion by increasing both heme pocket access and the structural mobility of the H-NOX domain. For example, in the heterodimer the N-terminal helix of the H-NOX domain (helix αA) is positioned so that its first 4 to 5 residues form part of the heme-binding pocket that contacts the heme (Fig. 5*C*). Tyrosine-2 is also part of a polar network of contacts that stabilizes the secondary structure elements lining the mouth of the heme-binding pocket, while also interacting with the sGCβ CC domain in the sGC heterodimer structure. HDX data show that Hsp90 binding to apo-sGCβ induces a large increase in the mobility of helix αA and a potential decrease in its secondary structure (Fig. 5*C*) (77). Other residues that line the heme pocket or help to mediate intradomain packing between subdomains of the sGCβ H-NOX also undergo an increase in solvent exposure upon Hsp90 binding to apo-sGCβ (Fig. 5*C*) (77). Several loops in the opening to the heme-binding pocket, which engage in intramolecular interactions with the CC domain in the heterodimer structures, may be similarly disrupted upon Hsp90 binding. Finally, disruption of intramolecular PAS-H-NOX domain interactions by Hsp90 may increase the lability of H-NOX helix αF, which contains the heme-binding axial residue His105 (Fig. 5*C*), further promoting heme access. Overall, we speculate that Hsp90 binding to apo-sGCβ disrupts HNOX-PAS-CC intramolecular interactions. In turn, this opens the apo-sGCβ tertiary structure, freeing H-NOX and removing potential steric blocks to heme pocket access and increasing the mobility of structural elements that line its heme pocket opening. Together, these changes would improve access for heme entry and ease heme transfer from GAPDH to apo-sGCβ.

## sGC inactivation

**Figure 6:**
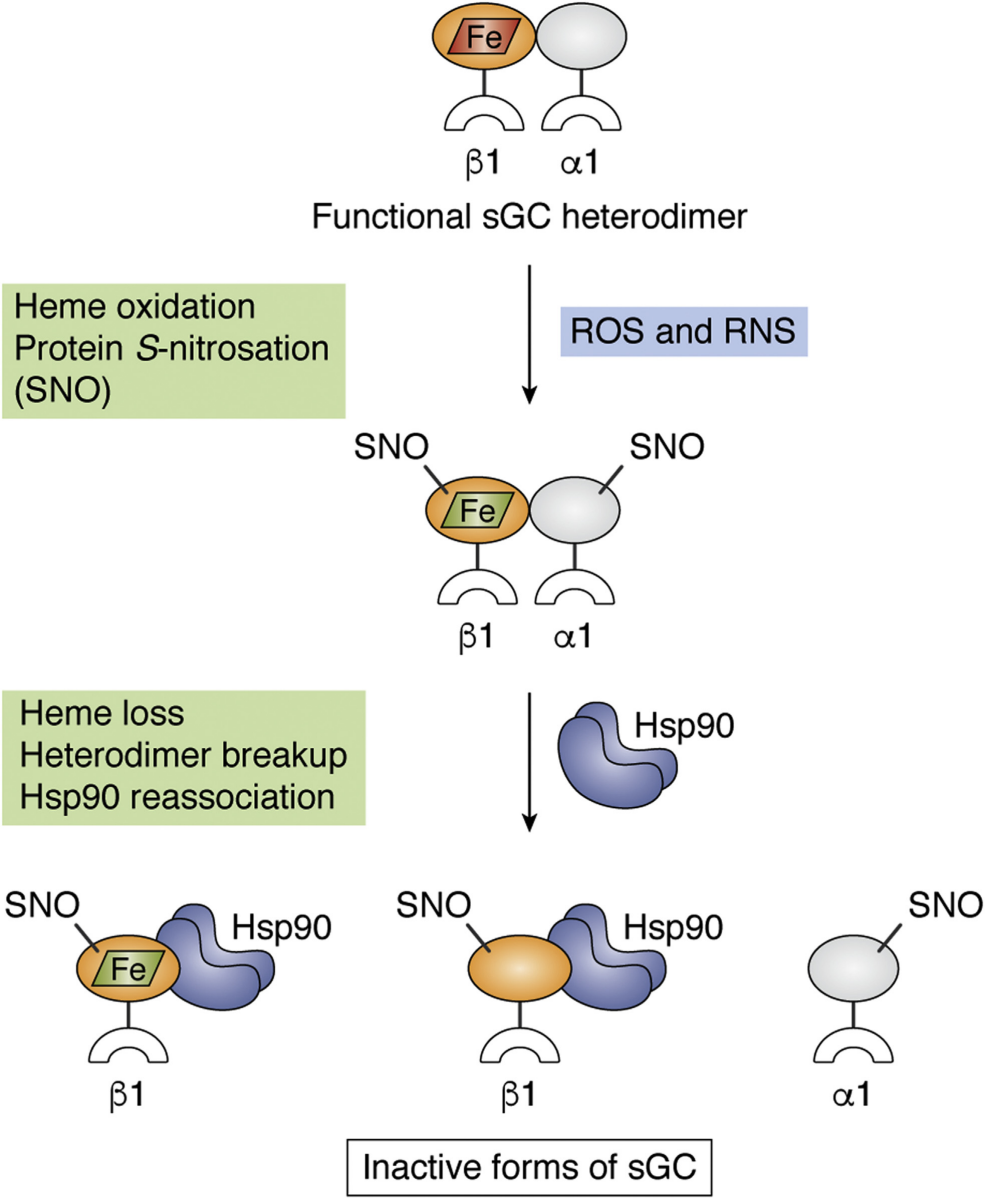
**sGC inactivation pathways and potential structural consequences.** When functional sGC heterodimer (top) is exposed to reactive oxygen and/or reactive nitrogen species (ROS and RNS) it can become unresponsive toward NO by undergoing oxidation of its ferrous heme to ferric (*red* and *green parallelograms*) and protein modifications like Cys S-nitrosation (SNO). These events alone or in combination may lead to breakup of the sGC heterodimer, heme loss, and/or rebinding of Hsp90 to the freed sGCβ1 subunit. NO, nitric oxide; PAS, Per-Arnt-Sim; sGC, soluble guanylate cyclase.

Inactivation of the sGC heterodimer is synonymous with development of insensitivity to activation by NO (31, 84, 85, 86). NO insensitivity can arise because of oxidation or loss of the sGC heme or upon covalent modification or oxidation of its Cys thiols. In some cases, sGC inactivation is associated with heterodimer dissociation. These mechanisms are noted in Figure 6 and discussed below.

### sGC heme oxidation and heme loss

Although sGC maintains its heme in the ferrous state (14, 18), heme oxidation is often invoked to explain the development of NO insensitivity in cells and tissues under oxidative stress (26, 86, 87). In cardiovascular and pulmonary diseases associated with increased oxidant stress, sGC heme oxidation may also contribute to the tissues developing resistance against NO-releasing vasorelaxants (31, 86). The xenobiotic chemical 1*H*-[1,2,4]oxadiazolo-[4,3-*a*]quinoxalin-1-one (ODQ) rapidly oxidizes the ferrous heme of sGC. ODQ is widely used to oxidize the heme of both purified sGC as well as sGC expressed in cells and tissues (35, 36, 86, 88). However, it is still unclear whether any native physiological or cellular species oxidize the sGC heme. Cell-generated reactive oxygen species and reactive nitrogen species such as H_2_O_2_ and peroxynitrite have been invoked as potential sGC heme oxidizers (68, 89, 90, 91, 92). In contrast, cytochrome b5 reductase isoform 3 (CYB5R3) catalyzes reduction of the sGC ferric heme (93). It is thus a challenge to understand the extent of sGC heme oxidation in biological settings under which both sGC and CYB5R3 are expressed. Overall, heme oxidation may be an important mechanism of sGC inactivation, but fundamental questions remain about when and by what molecular mechanisms its heme oxidation occurs under physiological conditions.

Heme loss is also widely invoked to explain the accumulation of inactive sGC (34, 86, 88) (Fig. 6). The first indication that sGC might be prone to heme loss was historically noted when the enzyme was first isolated. The investigators found that addition of heme increased the activity of the partially purified enzyme (94, 95, 96). Heme loss from the sGC heterodimer was suspected as the basis of this phenomenon. However, another explanation that takes current knowledge into account is that the tissues under study contained significant levels of immature heme-free sGCβ, which then formed newly active heterodimer upon heme addition. Indeed, the only heme-free sGC component that is definitively present in mammalian cells is the immature apo-sGCβ subunit in complex with Hsp90 (53, 76). While purified sGC heterodimer can be rendered heme-free by mild detergent treatment (97, 98), the existence of heme-free heterodimer in cells and tissues has not been widely demonstrated. A notable exception occurs when pharmacological agonists such as BAY 58 or BAY 60 are added to cells that express heme-oxidized or apo-sGCβ along with sGCα. In these cases, however, the agonist presumably either promotes dissociation of the oxidized heme (32) or directly binds within the heme pocket of apo-sGCβ and drives the formation of sGC heterodimers in which the “heme-free” sGCβ subunit now contains the agonist molecule in place of heme (53).

Because sGC displays high affinity toward ferrous heme (14, 18, 32, 34), a stepwise heme oxidation and loss mechanism is often invoked to explain how heme-free sGC may form in biological settings. Oxidation of the ferrous heme to ferric heme increases its dissociation rate from a negligible value up to rates ranging from 5 × 10^−4^ min^−1^ to 10^−2^ min^−1^, with the faster values being reported for truncated sGCβ proteins and the slower values being reported for full-length mammalian sGC (32, 33, 34). However, these rates are still very slow and imply that sGC maintains a relatively strong affinity toward bound ferric heme. While it is possible that the cellular environment somehow hastens loss of ferric heme from sGC, no specific mechanisms for such a process have been advanced so far. Addition of competitive heme-pocket binding agonists like BAY 58 caused a 5-fold increase in the rate of heme loss from purified ferric sGC (32). However, a similar agonist-induced heme loss has not been demonstrated yet for sGC when it is expressed in intact cells. Curiously, mild detergent treatment causes facile heme removal from sGC without irreversibly damaging the protein structure (97, 98, 99). This suggests that partial unfolding of sGCβ is a more effective way to remove the heme than is heme oxidation alone. Clearly, fundamental questions remain about how, when, and to what extent heme loss from sGC takes place in cells or tissues.

### Cys thiol modification and roles for NO and heme

sGC contains a greater number of Cys residues than expected based solely on its molecular weight (86). Site-directed mutation studies of sGC overexpressed in insect cells showed that many of the Cys residues are required for proper sGC maturation and function (100). Moreover, sGC thiol redox changes can regulate sGC activity both negatively and positively (84, 86, 101, 102, 103, 104).

When sGC-expressing cells are exposed to NO, the Cys residues of sGC often become S-nitrosated (Cys-NO) (Fig. 6). Cys nitrosation occurs whether NO is generated by cells or provided by an NO-releasing donor molecule (85, 105, 106, 107). The Cys-NO residues may further convert into S-oxides or disulfides, which potentially complicates interpretation of the effects of Cys nitrosation (106, 108, 109). Cys residues that undergo S-nitrosation are located in all four domains of the sGCα and sGCβ subunits (105). Although sGC inactivation develops concurrently with the buildup of Cys-NO residues, whether and how the specific S-nitrosation events may lead to sGC inactivation is still largely unclear. A chemical NO donor induced S-nitrosation of Cys residues 78, 122, and 214 in sGCβ and Cys 243 in sGCα. Moreover, site-directed substitution approaches showed that preventing S-nitrosation of Cys 122 in sGCβ and Cys 243 in sGCα mostly prevented purified sGC from developing NO insensitivity (84). In other studies, cellular NO production induced by receptor-driven events led to S-nitrosation of a different set of sGC Cys residues, potentially because of trans-nitrosation reactions (101, 102). In such cases, nitrosation at a distinct location (Cys 516) was linked to sGC inactivation (107). Together, these studies provided the first evidence that specific S-nitrosation events in sGC drive the development of NO insensitivity and inactivation, though potentially by multiple independent mechanisms. Clearly, further work is needed to understand the inactivation mechanisms at the molecular level.

Notably, the heme status of sGC may impact its Cys-NO modifications. Ferric sGC heme can engage in heme-catalyzed S-nitrosation of the Cys groups in sGC (33, 84, 101, 105, 110). This implies that the heme status of sGC (ferrous, ferric, or heme-free) helps to determine the extent and distribution of NO-induced Cys-NO modifications in sGC, which in turn could influence the mechanism and extent of NO-induced inactivation of sGC.

### Is heterodimer dissociation a common feature of sGC inactivation?

How heme oxidation, heme loss, or Cys modifications impact sGC structure has not often been considered. However, understanding the structural perturbations is likely key for understanding how sGC function becomes diminished in pathological settings.

Inactivating cell sGC by NO donor treatment leads to dissociation of sGC heterodimers and the association of liberated sGCβ subunits with Hsp90 (52, 53) (Fig. 6). Similar changes in sGCβ association were observed when sGC became inactivated in co-cultures of cells that express NO synthase and sGC (65) and in lung tissues in a mouse inflammatory asthma model where inducible NO synthase activity increases (65). In all cases, disruption of sGC heterodimers was associated with accumulation of Cys-NO modifications in sGCβ, but as noted, it is still unclear if or how these two events are causally linked. It is also unclear whether NO-driven inactivation of sGC precedes or coincides with heterodimer disruption. Moreover, it is still unclear if changes in sGC heme redox status or heme content, on their own, are sufficient to cause sGC heterodimer breakup and sGCβ–Hps90 association. Regardless, the studies so far have revealed that sGC heterodimer disruption is an overlooked but important structural consequence of NO-based sGC inactivation. Whether sGC heterodimer disruption represents a common general feature of its inactivation is certainly possible (Fig. 6) and should be investigated.

In sum, significant gaps remain in our understanding of sGC inactivation, particularly in biological settings. Such gaps include a paucity of information on how the sGC heme can become oxidized and lost from sGC and how commonly heme dissociation occurs. Moreover, we require a better understanding of what physical changes, including heterodimer breakup, occur when sGC is inactivated because of both NO-dependent and NO-independent heme oxidation, heme loss, or Cys thiol modifications in sGC.

## Recovery of inactive sGC

**Figure 7:**
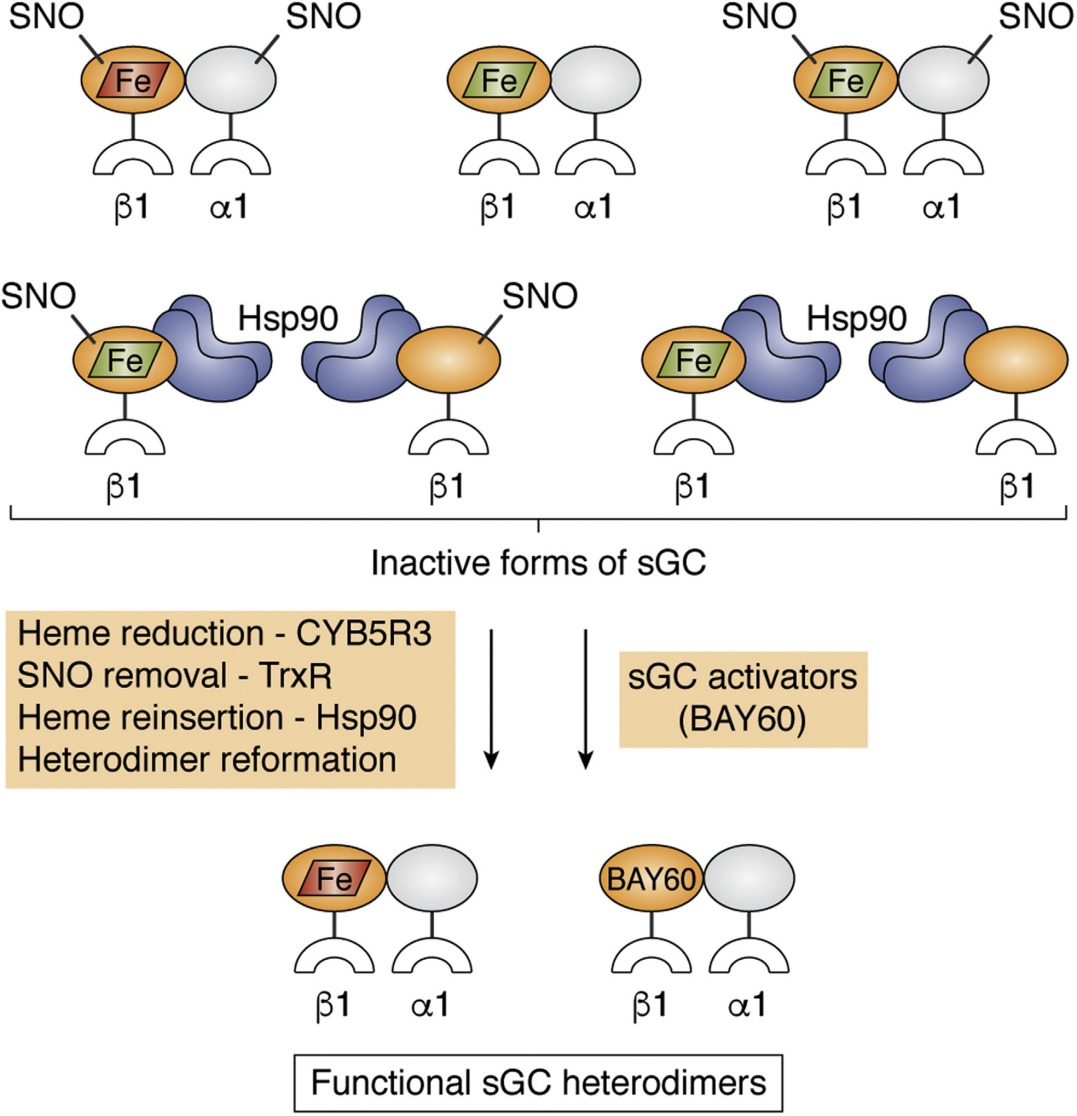
**Inactivated forms of sGC and potential recovery pathways.** Inactivated sGC may exist in at least seven forms in cells (*top*): A ferrous heme-containing (*red parallelogram*) heterodimer with Cys-NO (SNO) modifications, a ferric heme-containing (*green parallelogram*) heterodimer with or without SNO modifications, and dissociated forms in which a SNO-modified or SNO-free sGCβ subunit that either does or does not contain ferric heme is in complex with Hsp90. Conversion of the various inactive sGC forms to a functional sGC heterodimer may involve the steps and proteins and the small molecules noted in the *colored boxes*. sGC, soluble guanylate cyclase.

A number of cellular pathways have the potential to return inactivated sGC to its active form. As illustrated in Figure 7, recovery mechanisms include reduction of the sGC ferric heme, reversal of sGC Cys modifications, heme reinsertion into sGCβ, and reformation of the heterodimer. Below, we discuss these processes and their relationships to each other. Given that we understand relatively little about sGC recovery, the discussion is largely speculative with the exception of a few important examples.

### sGC recovery versus removal

Conceptually, sGC recovery is only important if the recovery processes compete effectively with removal of inactivated sGC in cells. This competition has not been studied directly. Studies on the *in vivo* expression levels of sGC subunits have indicated a complex relationship. For example, sGCα1-knockout mice expressed reduced levels of sGCβ in their tissues, and sGCβ knockout mice expressed undetectable levels of sGCα despite having normal mRNA levels (111, 112). Because the absence of one sGC subunit was enough to diminish or eliminate the buildup of the partner subunit, this work suggests that heterodimer formation may be mutually beneficial for the expression and stability of either sGC subunit. In experiments where knockout of smooth muscle–specific sGCβ expression was induced in mice, the preexisting sGCβ and sGCα proteins in tissues were found to exhibit fairly long half-lives of 3 to 4 days (113). sGCβ and sGCα proteins expressed in cell culture systems have relatively long half-lives (23, 47, 87, 114, 115). In mammalian cells under normal culture conditions, sGCβ accumulation or stability are largely unaffected by whether the subunit receives heme during its maturation or whether sGCα is co-expressed (53). It is interesting to consider whether Hsp90 binding protects apo-sGCβ from degradation, given that Hsp90 inhibitors hasten sGCβ disappearance in cells (61). Unspecified damage to sGC because of its inactivation induced by inflammation, immune stimulation, or by exposure to oxidants or NO was reported to decrease sGCα or sGCβ levels over fairly long timescales (12–24 h) (23, 42, 116, 117, 118). These reductions in sGC subunit levels may have been because of increased rates of proteolytic removal, but this possibility was not directly tested. Likewise, Hsp90 inhibition (61) as well as heme oxygenase 1 induction (71, 119) each lower sGCα and/or sGCβ activity and/or expression levels over a 24 h time period. In contrast, agonists like BAY 58 stabilize sGC protein expression levels (48, 88, 120). The mechanisms for regulation of intracellular sGC levels by any of these perturbations are unclear. A better understanding of these factors will provide critical insights into the relative importance of the sGC recovery mechanisms that are discussed below.

### sGC ferric heme reduction

Because heme oxidation is the simplest way to inactivate sGC, its reduction of back to ferrous represents the simplest mechanism for recovery (Fig. 7). Given that sGC stabilizes its bound heme in the ferrous state, the need for an enzyme dedicated to sGC heme reduction has been controversial. However, recent studies in animal models and cell culture provided convincing evidence that CYB5R3 functions, at least in part, to maintain the sGC heme in its ferrous state (93). For example, rat aortic smooth muscle cells treated with ODQ (to inactivate sGC) recovered NO sensitivity within 60 min after ODQ removal (93). This temporary inactivation resembles the reversible sGC inhibition by ODQ first observed in cerebellar slices (36). Such experimental evidence argues that cells and tissues contain a system to catalyze sGC ferric heme reduction. Knockdown of CYB5R3 expression in the rat aortic smooth muscle cells increased their levels of NO-unresponsive sGC (93). Experiments with the purified proteins showed that CYB5R3 was able to reduce the sGC ferric heme, albeit slowly, in a reaction that depended on NADPH and a direct protein–protein interaction (93). In a mouse model of sickle cell disease, CYB5R3 knockdown also enhanced the development of NO insensitivity of vascular sGC (121). Together, these findings argue that sGC heme oxidation occurs in oxidative or inflammatory settings and that heme reduction mediated by CYB5R3 is functionally significant for sGC recovery of function. It remains unclear which structural states of ferric sGC, such as heme-oxidized sGC heterodimer *versus* Hsp90-associated or free ferric sGCβ subunit, are effective substrates for CYB5R3 in cells (Fig. 7). In any case, sGC ferric heme reduction by CYB5R3 represents a plausible continuous cellular sGC recovery process that functions to maintain sGC NO sensitivity and signaling function.

### Recovery of sGC Cys thiols

Cys residues in sGC that become oxidatively modified or S-nitrosated can be returned to their free thiol forms by thioredoxin-1 and thioredoxin reductase (TrxR) (Fig. 7). Both enzymes are implicated in maintaining sGC in its NO-responsive state. For example, TrxR inhibition diminishes sGC activity and increases sGC S-nitrosation in cardiovascular tissue (122). Covalent interactions of Cys residues in sGC with thioredoxin-1 or with protein disulfide isomerase were observed and implicated in recovery of sGC thiols (123, 124). Regeneration of sGC Cys thiols may even allow them to recover their ability to participate in NO-driven activation of sGC (102). On the other hand, there is evidence that recovery of maximum sGC activity involves an oxidation of vicinal Cys thiols to the disulfide (101, 125). In sum, Cys thiol redox processes appear to be complex in sGC but important for its recovery, and further investigation should improve our understanding.

### Heme reinsertion into sGC

Heme loss from sGC is likely more difficult to repair than is heme oxidation. However, based on what we know about sGC maturation, cells have mechanisms in place to reinsert new heme into inactivated apo-sGC if it forms, particularly if the apo-sGCβ binds to Hsp90 after heme loss (Fig. 7). The Hsp90 interaction may help to coordinate heme delivery by GAPDH and aid heme insertion into the apo-sGCβ subunit, much as during sGC maturation. Such a recovery mechanism requires the sGC heterodimer to dissociate after heme loss so that the apo-sGCβ subunit can bind to Hsp90 (53, 70, 76) (Fig. 7). This structural change remains to be demonstrated. On the other hand, it is possible that heme loss leads sGCβ to be targeted for degradation in the cell. Hsp90 may also play a role in directing the sGCβ for removal (61, 126). These mechanisms remain to be explored.

### sGC recovery can be driven by small molecule sGC agonists

An ability to rescue inactivated sGC through pharmacologic means has already been demonstrated. It is well-known that sGC activator agonists such as BAY 60 or BAY 58 reactivate sGC that has become inactivated because of heme oxidation or presumed loss (47, 86). In such cases, the agonist is thought to either displace the oxidized heme (32, 34, 88) or replace the lost heme. When BAY 60 was administered to cells containing NO-inactivated sGC, it promoted sGC heterodimer reformation as part of the recovery process and did so independent of Hsp90 (53). Notably, unlike BAY 60, the sGC stimulator BAY 41 did not promote sGC heterodimer reformation or recovery (53). This implies that BAY 60 binding in the sGC heme pocket triggers its recovery mechanisms. Whether BAY 60 also promoted removal of any Cys–NO modifications from the NO-inactivated sGC was not investigated, but its mechanism of action may potentially also include the reversal of such modifications. In any case, small molecule sGC activators such as BAY 60 or BAY 58 are remarkably powerful tools to drive recovery of sGC that has become inactivated because of excess NO or oxidative stress, even after heterodimer breakup has occurred. Indeed, the current data suggest that these agonists might be capable of recovering sGC in all of its inactive forms (Fig. 7).

### Further thoughts on sGC recovery mechanisms

Most cells already express proteins that support sGC heme reduction (CYB5R3), recover S-oxidized or S-nitrosated protein thiols (thioredoxin-1, TrxR), or aid in heme insertion reactions (GAPDH, Hsp90). These proteins may function continuously in the background to recover sGC that becomes inactivated because of heme oxidation, loss, or thiol modifications. However, their effectiveness in a given circumstance would depend on their activity levels in the cell relative to the rate of accumulation of sGC damage. When the rate of sGC damage exceeds the cell's repair rate, a buildup of modified, inactive sGC forms is expected to occur. Overloading of cell repair capacities may explain how Cys-nitrosated sGC builds up in cells and tissues undergoing prolonged NO exposure (65, 101) after an initial period in which such modification is not seen and the NO exposure is helpful (65), and may also explain why sGC dysfunction increases during chronic oxidative stress (127). Recovery may also depend on which of the inactive sGC forms represent suitable substrates for the repair proteins *versus* the forms that actually accumulate in cells (Fig. 7). For example, buildup of inactive sGC heterodimers *versus* buildup of the dissociated sGCβ–Hsp90 complexes would present distinct challenges to the recovery processes. A related issue is the scope of the Hsp90 interaction with the different forms of inactive sGC and whether the Hsp90 interaction plays a passive or active role in recovery by heme reduction, heme reinsertion, or Cys–NO removal and thiol reformation. These questions should now be addressed.

## Conclusions and perspectives

This review strove to highlight aspects of sGC that receive comparatively less attention. Two fundamental concepts emerge: (1) In the course of its lifetime sGC can exist in more forms than what was typically presumed; and (2) Hsp90 plays substantial roles that differ from what had previously been surmised. A hallmark of the first concept is the remarkable propensity of sGC to accumulate in its immature heme-free form in cells and tissues. This provides cells with an opportunity to utilize the mechanisms of sGC maturation in a dynamic way to regulate their level of sGC catalytic activity. Indeed, that NO can promote sGC maturation (53, 65) already hints that this strategy is in use. As an example of the second concept, it is remarkable that Hsp90 impacts sGC at the two opposite ends of its lifetime, rather than impacting sGC in its catalytically active heterodimeric form. Hsp90 interacts on one hand with the immature apo-sGCβ to enable sGC maturation, and on the other hand interacts with oxidatively-damaged sGCβ after heterodimer breakup, perhaps to enable its repair or instead direct its demise. In this way, Hsp90 first acts like a governess to apo-sGCβ in its youth and then as a nurse to sGCβ in its old age, and the only chance for sGCβ to partner with sGCα comes during its productive middle lifetime.

On a broader level, it is interesting to ask if sGC is just a one-off example of these behaviors or is instead herald of a more general condition in which cells and tissues naturally build up overlooked but significant levels of heme proteins in their heme-free immature forms. Indeed, evidence shows that this does in fact occur for other hemeproteins including indoleamine or tryptophan dioxygenases, both when they are expressed naturally in liver (128) or are expressed in cultured cells (129), NO synthases expressed in cultured cells (130), and for myoglobin and hemoglobin β and γ in cells of both erythrocytic and nonerythrocytic lineage (59, 60). Notably, in a majority of these cases the apo-hemeprotein subpopulations were found associated with Hsp90, just like for apo-sGCβ. We therefore suspect that sGC is just one clearer example of a more general and underappreciated phenomenon in which a significant proportion of many different hemeproteins are naturally kept in their heme-free forms, often in association with cell chaperones. To what extent these “partly anemic hemeproteins” exist in health or disease, which hemeproteins do or do not participate, and whether NO can act more broadly to influence their heme content and maturation, are fundamental questions that remain to be explored.

Many questions still remain about sGC itself. For example, how do its maturation processes relate to its intracellular localization (46, 131)? How is the sGCα subunit handled within cells? Is it stabilized while awaiting heme insertion into the apo-sGCβ-Hsp90 complex? Is there a specific sGCα chaperone protein? After all, this is the case for hemoglobin-α, which requires its own chaperone called alpha hemoglobin stabilizing protein (132), whereas hemoglobins β and γ are Hsp90 clients (59) like apo-sGCβ.

Given how prevalent sGC inactivation occurs in disease, it is surprising how little we know about the molecular basis for its genesis or the attendant recovery processes. Which of the possible inactive forms of sGC build up in cells and tissues, and under what circumstances? During development of NO insensitivity, what cell-derived molecules oxidize the sGC heme? What are the actual impacts of the various cysteine modifications to sGC? To what extent do any of these changes drive sGC heterodimer dissociation? Regarding sGC recovery, it will be important to determine how the recovery enzyme systems discussed herein (CYB5R3, Thioredoxin 1, TrxR), related cell redox systems, and Hsp90 are involved, and under what circumstances. It is also remarkable that sGC activators like BAY 60, seemingly on their own, can drive recovery of inactive sGC through a reformation of an active heterodimer. The basis for this rescue needs to be understood and leveraged for therapeutic goals.

## Conflict of interest

The authors declare that they have no conflicts of interest with the contents of this article.
